# A Method for Segmenting Medicare Expenditures to Inform Cost Effective Care Delivery for Older Adults

**DOI:** 10.5334/egems.272

**Published:** 2019-07-16

**Authors:** Christopher Crowley, Tyler Kent, Liane Wardlow, Martha Twaddle

**Affiliations:** 1 West Health Institute, US; 2 Northwestern University Feinberg School of Medicine, US

**Keywords:** Medicare, Delivery of Health Care, Health Care Cost, Health Data, Health Services Research

## Abstract

**Introduction:**

Faced with growing populations of older, medically complex patients, health systems are now incentivized to deliver cost-effective, high-value care. We evaluated a new method that builds upon existing Medicare spending concentration studies to further segment these expenditures, revealing use patterns to inform care redesign.

**Methods:**

We obtained monthly Medicare expenditure data and derived baseline comparison data using typical methods for identifying a yearly high-cost subpopulation. We then applied the new methodology, ordering monthly patient expenditures from highest to lowest to more extensively segment the baseline data. Our evaluation examined the following within the new more extensive segmentation: monthly expenditure distribution, corresponding patient counts, and occupancy of specific patient subgroups within the extended segmentation of baseline data.

**Results:**

Compared to the baseline data, we found further spending concentration, with 16.7 percent of high-cost patients being responsible for about two-thirds of baseline expenditures. The remaining 83.3 percent of the high-cost subpopulation exhibited lower spending, collectively accounting for about one third of baseline expenditures. Additionally, we found that unique patient subgroups occupied different segments over time, with specific subgroups comprising 8.3 percent of the study subpopulation patients migrating into and out of each highest spending segment, accounting for almost half of monthly baseline expenditures.

**Conclusions:**

With monthly health care expenditures concentrated among small numbers of migrating patients, our evaluation suggested potential cost-effectiveness in tiered care delivery models, where small subgroups receive direct, active care interactions, while the remainder experience surveillance-level care, designed to both address ongoing medical needs and to detect emergent migration.

## Introduction

Throughout much of the world, health systems are facing challenges associated with growing populations of older, more medically complex patients [1]. In the United States (US), for example, the number of Americans age 65 and older is expected to rise to almost 100 million by 2060, ultimately representing as much as one quarter of the total population [2]. Because budgetary resources are often limited, it is necessary to deliver cost-effective, high-value care for this aging population. Within the Medicare health insurance program, which covers older Americans, there are imperatives to shift away from a legacy of expensive volume-based incentives to support more value-based care delivery [3]. Studies of Medicare expenditures can shed light on utilization patterns intrinsic to people in the U.S. 65 years and older, as well as providing context for demonstrations of comparative cost-effectiveness. Such characterizations could specifically inform approaches for designing health systems to more efficiently meet the needs of America’s growing population of older adults.

Historically, studies of Medicare expenditures have highlighted spending concentration, in which high-cost beneficiaries disproportionately drive overall yearly spending [4,5,6,7,8]. These well-established concentration patterns have been used as a basis for care redesign. Specifically, providers have considered potentially more efficient care delivery models that target high-cost beneficiaries [9,10] by adding infrastructure [11] and/or human resources in the form of care coordinators or disease managers [12]. Results from numerous programs including an extensive series of Medicare demonstrations indicate that these additional resources were sometimes effective at reducing high-cost services (e.g. hospitalizations), provided they developed substantial, direct interactions between care coordinators, patients and their physicians [12]. However, most of these programs were not cost-effective overall, after accounting for the fees associated with providing the additional resources [12]. Thus, more extensive, in-depth characterizations of Medicare spending may inform care redesign by providing a clearer basis for targeting of care coordination and other resources.

In several recent studies, researchers identified more extensive segmentations of expenditures for high-cost Medicare beneficiaries [13,14,15]. Based mainly on clinical criteria, certain patient subgroups (e.g., characterized by frailty) identified from within the top 10 percent of Medicare spenders were found to occupy distinctly higher spending segments [13,14,15]. In the present study, we develop more extensive spending segmentation for these same high-cost beneficiaries, but we do not attempt to delve further into clinical considerations. Instead, we evaluate a new segmentation method that indicates the extent to which certain Medicare patient subgroups have high monthly health care expenditures and who often migrate in and out of the highest spending segments on a monthly basis. The hypothesis underlying our investigation was that indiscriminately or persistently providing care coordination or other resources—even within the top 10 percent of Medicare spenders—could inadequately target care delivery to the subgroups who may migrate at any time to occupy the highest spending segments. By quantifying attributes of monthly spending migration for these patient subgroups, the new approach could complement existing clinical segmentation methodologies and inform better targeted care, delivered when and where it is most needed. Although initially developed using Medicare data, this new methodology can be adapted to evaluate expenditure data from other programs facing similar demographic trends and challenges.

## Methods

### Study Aim

Our aim was to build upon existing Medicare spending concentration studies by investigating a new method for more extensively segmenting expenditures, to answer three specific study questions:

1. What is the monthly distribution of aggregated spending resulting from the new, more extensive segmentation?
2. What is the corresponding monthly distribution of aggregated patient counts?
3. What is the occupancy of specific patient subgroups within the segmentation of aggregated patient counts and expenditures?

### Study Design and Setting

To answer these study questions, we designed a study using historical Medicare claims data for older adults in the US. Baseline spending data comprising of monthly expenditures for a 10 percent high-cost subpopulation were obtained, beginning with typical methods for characterizing spending concentration. We then calculated monthly spending order index assignments to further segment monthly spending and population counts for comparison against the original baseline data. Finally, occupancy of specific patient subgroups throughout the resulting segmentations were characterized. Following the investigation, implications for cost-effective care delivery and context for certain related demonstrations are presented.

To analyze both baseline and segmentation data, Medicare spending data were obtained from a national standard 5 percent random sample of beneficiary claims from 2012. These expenditure data included so-called Standard Analytical Files (SAFs) for both institutional claims (e.g. Inpatient, Outpatient, Skilled Nursing, Home Health and Hospice) and non-institutional Carrier claims. A denominator file, containing anonymized unique patient identifiers (DESY_SORT_KEYs) was also obtained. Data associated with Medicare Advantage (Part C), Medicare Part D (drugs), and Durable Medical Equipment (DME) were not used. All data were procured as a Limited Data Set (LDS) from the Research Data Assistance Center (RESDAC) [16] and secured using a HIPAA-compliant cloud-based data storage service (Amazon Web Services) [17].

### Initial Population

The initial population included Medicare beneficiaries age 65 years and older who had continuous Medicare Part A and Part B enrollment over the 2012 study period. Patients who passed away during 2012 were included in the analysis, provided they had continuous Part A and Part B enrollment up until death. Medicare patients having no claims during 2012 were excluded from the study. These criteria yielded an initial population of 1,222,402 Medicare beneficiaries representing $13.88B in total yearly spending.

### Analysis: Baseline Spending Data

All data were processed using a Structured Query Language (SQL) application (Amazon Redshift) [18]. Total yearly expenditures for each beneficiary were first calculated by summing all claims in the 2012 LDS files. Using the resulting yearly spending values, we generated a list of all beneficiaries, ordered by yearly expenditures from the highest to lowest values. From this ordered list, the top 10 percent (n = 122,241) were identified as the high-cost subpopulation used to study the present method. Yearly expenditures for these beneficiaries added up to $8.03B, representing a disproportionate concentration of 58 percent of the yearly expenditures of $13.88B for the initial population described above. We then obtained estimates of total monthly expenditures for each member of this high-cost subpopulation, by adding up patient-level expenditures in the respective calendar months based on the “claim through” date in each claim contained in the SAFs. Months during which a beneficiary had no expenditures were assigned zero spending, so that every member of the study subpopulation had a defined spending value for every month. A summation of expenditures aggregated over all the beneficiaries in high-cost subpopulation was then performed, grouping and ordering sums over the 12 separate months [19,20]. The resulting monthly aggregated expenditures for the top 10 percent of spenders were defined to be the baseline spending data for the high-cost study subpopulation.

### Analysis: Segmented Data

**Figure 1:**
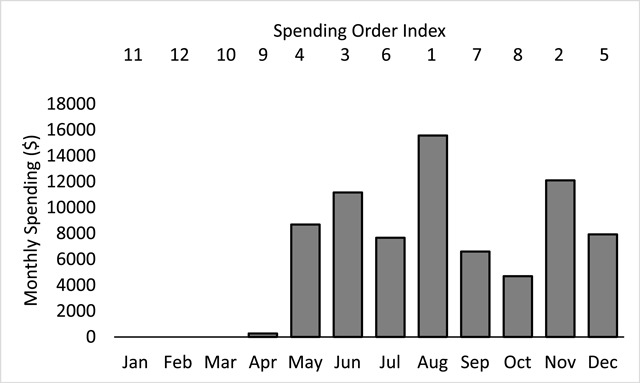
Monthly spending for a single representative Medicare beneficiary chosen from the top 10 percent of spenders representing the high-cost study subpopulation. Monthly spending order index assignments are indicated above each of the plotted monthly spending values. Unique spending order indices are assigned over all 12 months, even in cases of equal valued monthly spending.

The segmented data were derived from the same high-cost subpopulation (n = 122,241) used to calculate the baseline spending. For each of these top 10 percent of spenders, monthly expenditures were first assigned a unique supplemental spending order index identifying the highest to lowest spending values for each month. Figure 1 illustrates such spending order index assignments for the monthly expenditures from a single beneficiary randomly chosen from the high-cost subpopulation. Importantly, the spending order assignment provided a unique index value even when encountering two different months having equal expenditures, for example, when multiple months exhibited zero spending values [20]. A summation of expenditures aggregated over all beneficiaries in the high-cost subpopulation was again performed, this time grouping and ordering over both the 12 separate months and the 12 separate spending order indices [19,20]. The resulting data comprised of 144 separately aggregated values, representing the segmented spending data needed to answer the first study question above. In addition, the number of beneficiaries contributing to each of the 144 separate segments was calculated for each month, using an aggregated count of all high-cost beneficiaries, again grouping and ordering over both the 12 separate months and the 12 separate spending order indices [19,20]. The resulting segmented beneficiary counts provided the answer to the second study question above.

Finally, to answer the third study question, we investigated partitioning properties resulting from the spending order index assignment to understand how unique subgroups of beneficiaries occupy different segments of both aggregated beneficiary counts and spending values. Specifically, we isolated subgroups of beneficiaries corresponding to the highest segment in each month by selecting all DESY_SORT_KEYS having the highest spending order index for each of the respective months. We then tracked and plotted the occupancy of these isolated subgroups throughout each of the 144 patient count and spending segments described above.

## Results

### Baseline Spending Data

**Figure 2:**
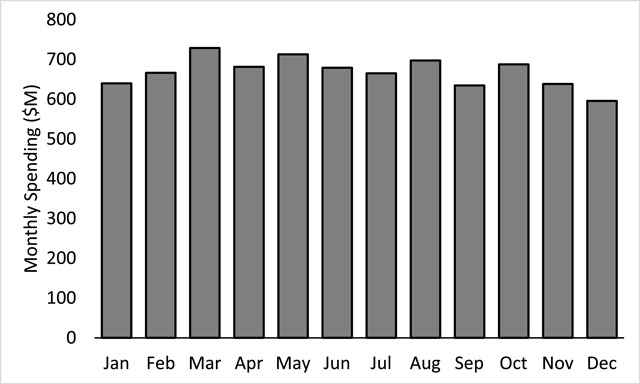
Baseline spending comprising aggregated monthly expenditures (Millions $) for the top 10 percent of spenders (n = 122,241). Average monthly spending for this high-cost study subpopulation was $669M with a standard deviation of $36M for a total yearly spending value of $8.03B.

The aggregated monthly expenditures defined to be baseline spending data for the high-cost subpopulation are illustrated in Figure 2. The total yearly expenditures of $8.03B for these top 10 percent of spenders were distributed relatively evenly throughout the 2012 calendar at a rate of $669M per month with a standard deviation of $36M.

### Segmented Data

**Figure 3:**
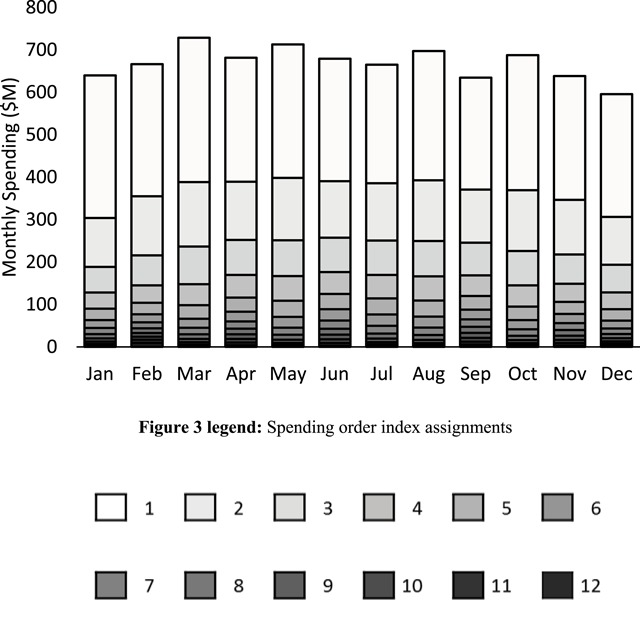
Segmented spending data comprising of 144 separately aggregated spending values. Columns correspond to the indicated months and stacked data values correspond to the indicated spending order index values. Greyscale shading is used to represent the range of spending order indices with the highest segment having an index value of 1. Spending segmentation revealed further concentration within the baseline values, with almost two thirds of monthly expenditures accounted for by the highest and second highest spending segments.

The segmented expenditure data comprising of 144 separately aggregated spending values are shown in Figure 3, with columns representing the months and stacked data values used to indicate the separate spending order indices. Greyscale shading is used to represent the range of spending order indices with the highest segment having an index value of 1. Compared with the baseline spending data in Figure 2, Figure 3 shows that the original monthly spending for the top 10 percent of Medicare spenders has been separated into distinct, non-overlapping spending segments. These new spending segments exhibited further spending concentration, beyond the concentration of yearly spending intrinsic to the high-cost subpopulation and typically uncovered via normal analyses. Specifically, the highest segment in Figure 3 accounted for an average value of $302M—nearly half of the average baseline monthly spending of $669M. Similarly, the second highest segment accounted for an average value of $134M, representing about one fifth of the average baseline monthly spending. Together, the average first and second highest segments accounted for $436M—almost two thirds of the average baseline monthly spending for the top 10 percent of spenders. All other segments (e.g. from third to twelfth) collectively accounted for only about one third of the total average baseline monthly spending. The standard deviations for the first and second segments were $22M and $11M respectively, indicating relative uniformity in terms of month-to-month variation in the segmented spending shown in Figure 3.

**Figure 4:**
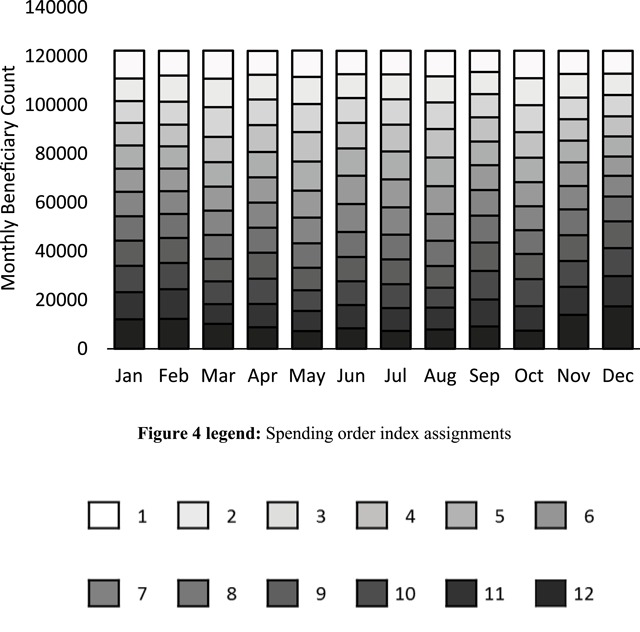
The number of beneficiaries contributing to each of the 144 aggregated beneficiary count segments, with columns representing months and stacked data values using the same greyscale shading used in Figure 3 to indicate the corresponding spending order indices. In any given month, the high-cost study subpopulation comprising of n = 122,241 beneficiaries was divided into 12 segments containing an average count of 10,187 beneficiaries with a standard deviation of 1283.

The number of beneficiaries contributing to each of the 144 aggregated beneficiary count segments is shown in Figure 4, with columns representing months and stacked data values using the same greyscale shading to indicate the corresponding spending order indices. Within any given month, the total count of n = 122,241 for the high-cost subpopulation was separated into twelve distinct, nonoverlapping beneficiary count segments, each having an average count of 10,187 beneficiaries (standard deviation 1283).

Thus, each of the spending segments shown in Figure 3 corresponded to a relatively uniform beneficiary count segment, totaling about one twelfth, or 8.3 percent of the high-cost subpopulation. Given that the highest segment for any given month accounted for almost half of that month’s spending, it follows that 8.3 percent of the high-cost subpopulation specifically corresponded to each month’s highest segment and therefore accounted for almost half of that month’s baseline spending. Similarly, the next distinct 8.3 percent of the same high-cost subpopulation corresponded to the second highest segment, so that a total of about 16.7 percent of the high-cost subpopulation—those beneficiaries corresponding to the highest and second highest segments—accounted for almost two thirds of baseline spending in each month. The remaining 83.3 percent of the high-cost subpopulation cumulatively accounted for just over one third of baseline expenditures in each month.

**Figure 5:**
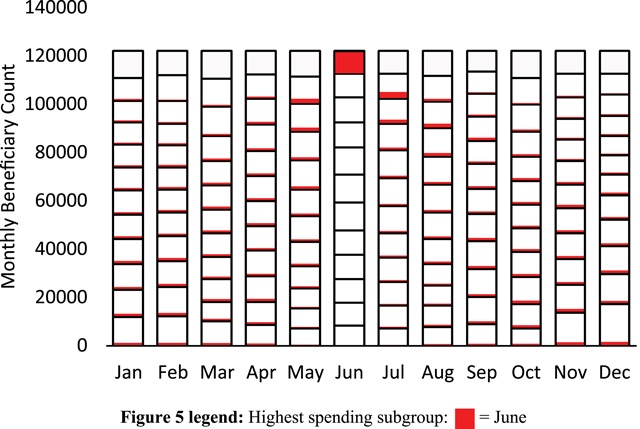
Aggregated counts of beneficiaries for the isolated subgroup occupying the highest segment in the month of June. Color-coding (red) is used to designate the contribution of the isolated June subgroup to the count of beneficiaries in each segment. No member of the isolated June subgroup occupied any lower segments in June. Similarly, no member of the same June subgroup occupied the highest segment in any month other than June. The total count of beneficiaries within the isolated June subgroup remained constant throughout the year.

To investigate how unique subgroups of the high-cost subpopulation migrated throughout the segmentations of both aggregated beneficiary counts and spending values, we first isolated the subgroup of beneficiaries occupying the highest segment in a single month. We began with the month of June, to investigate occupancy patterns prior and subsequent this highest segment month. The occupancy of this particular June subgroup throughout the segmentation of beneficiary counts is shown in Figure 5. In Figure 5, the segment boundaries used in Figure 4 are preserved. However, the grey scale indication of segment indices has been replaced by a unique color-code (red) to indicate the count of beneficiaries from the isolated June subgroup in all 144 segments. In Figure 5, the highest segment for June was fully occupied by the red-coded subgroup. However, no member of this same subgroup simultaneously occupied any other segments in June, as indicated by the white spaces in all segments below the highest June segment. Similarly, white spaces to the right and left of the highest June segment indicate no occupancy in these highest segments for any months other than June. The partial white spaces in all other segments in Figure 5 indicate partial occupancy, with the isolated June subgroup distributed throughout lower segments in the months prior and subsequent June. More specifically, beginning in the months prior, the June patient subgroup migrated up from lower segments to exclusively occupy the highest segment in June, and then migrated back down to the lower segments in the subsequent months. In Figure 5, the total count of beneficiaries for the June subgroup was the same every month, despite changing occupancy and distribution among different beneficiary count segments.

**Figure 6:**
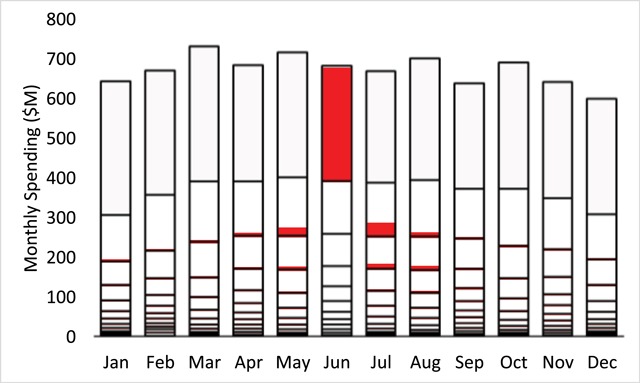
Aggregated monthly spending (Millions $) for the isolated subgroup occupying the highest segment in the month of June. Red color-coding is again used to designate occupancy and migration of the isolated June subgroup. Aggregated spending for the isolated June subgroup was highest in June while occupancy and migration in months prior and subsequent June were accompanied by lower total spending.

The occupancy of this same June subgroup throughout the segmentation of aggregated spending is shown in Figure 6. The same red color-code in Figure 6 indicates occupancy and migration patterns similar to those in Figure 5. Specifically, full occupancy in the highest spending segment is indicated by the red color code filling the entire highest segment in June, while white spaces indicate no occupancy and therefore no spending in both the lower June segments and in the highest segments in months other than June. Partial occupancy and therefore partial segment spending is similarly represented by partial white space in all other segments. Total spending for the June subgroup was not the same every month. Specifically, in the months prior and subsequent June, the aggregate spending of the isolated June subgroup was lower, in addition to being distributed among the various lower segments.

**Figure 7:**
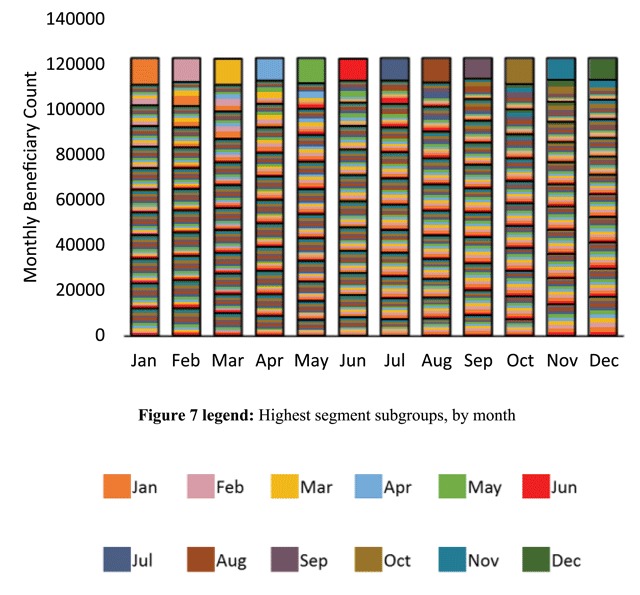
Aggregated counts of beneficiaries for the isolated subgroups occupying the highest segment in each respective month. A unique color-coded subgroup fully filled each month’s highest segment and partially filled lower segments, prior and subsequent the highest segment month.

**Figure 8:**
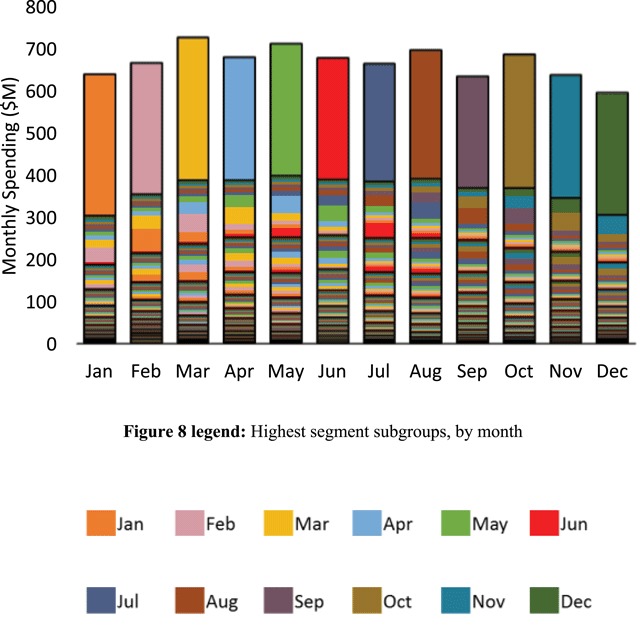
Aggregated spending (Millions $) for the isolated subgroups occupying the highest segments in each respective month. The same unique color-code used in Figure 7 designates the contribution of the respective isolated subgroup to the aggregated spending in each segment. In each month, a unique subgroup comprising approximately 8.3 percent of the high-cost study subpopulation accounted for almost half of that months spending.

We next isolated all other highest segment subgroups for the other months in addition to June. Figure 7 represents the resulting aggregated patient counts and Figure 8 represents the resulting aggregated spending. In addition to June, Figures 7 and 8 use unique color codes to identify each of the isolated subgroups for all other months. In both figures, the respective highest segments were fully occupied by each isolated subgroup, as evidenced by the unique single-color code that fully fills each of the highest segments. Migration and partial occupancy in lower segments in the months prior and subsequent the months corresponding to the isolated subgroups is reflected in the multiple colors filling each of the lower segments. Note that Figure 8 exhibited the same spending segmentation shown in Figure 4. However, Figure 8 further indicates that the relatively uniform spending segmentation for each month in Figure 4 represented a type of dynamic equilibrium, in the sense that unique patient subgroups migrated in and out of the highest spending segments each month.

In summary, the new segmentation method separated the original baseline monthly spending for the top 10 percent of spenders into distinct, non-overlapping spending segments. Two-thirds of monthly baseline spending was associated with only 16.7 percent of this high-cost study subpopulation. The remaining 83.3 percent of the study subpopulation was associated with only one third of monthly baseline spending. Additionally, 12 isolated subgroups—each comprising just 8.3 percent of the high-cost study subpopulation—migrated into and out of the highest spending segments each month, and fully accounted for almost half of baseline monthly spending.

## Discussion

Our findings of both monthly spending concentration as well as migration of subgroups responsible for much of that spending provide support for the hypothesis underlying the present investigation. Specifically, with 83.3 percent of the top 10 percent of spenders occupying lower spending segments most of the time, indiscriminately adding persistent care coordination or other resources could result in resources being expended out of proportion with the actual needs of most high-cost patients at any given time. As a corollary, with almost half of monthly baseline spending resulting from migrating subgroups comprising just 8.3 percent of the top 10 percent of spenders, opportunities for impacting these subgroups may be few and far between. More specifically, the highest segment subgroups were replaced every month, so waiting to provide active care coordination or other resources until such subgroups emerge could result in missed opportunities to provide direct, substantial interactions when and where they are most needed.

Further support for the underlying hypothesis as well as the above corollary may be found through descriptions of demonstration programs where care redesign aligns with the patterns of spending concentration and migration identified in the present study. Such aligned care delivery is necessarily both tiered and adaptive in nature, in accordance with the dynamic migration of subgroups illustrated in Figure 8. As an example, in the Massachusetts General Hospital (MGH) Care Management Program [21], skilled care managers were each assigned to about 200 high-risk Medicare patients at a time, with about 15 percent of the assigned patients (n = 30) receiving active care at any one time. The other 170 patients occupied a lower tier of routine surveillance [21]. The assignment of 15 percent of the population to an active tier of care coincides approximately with the 16.7 percent of high-cost beneficiaries occupying the highest and second highest spending segments identified through the present method. In terms of supporting the lower-tier surveillance group of beneficiaries, the MGH team describes using a dashboard that allowed tracking, on a weekly basis, to identify potentially emergent trends in utilization [21]. Overall, the MGH Care Management Program was associated with improved cost-effectiveness, both in an early Medicare demonstration [12], and more recently in an extension through the Partners Healthcare Pioneer ACO [22]. More specifically, in the Medicare Care Management Program for High Cost Beneficiaries Demonstration, the MGH program achieved the highest net reduction in Medicare spending when compared to thirty-four related programs evaluated across six demonstrations, with most programs achieving no such reduction [12]. In the Pioneer ACO continuation, the program continued to demonstrate cost-effectiveness with a net reduction in Medicare spending of six percent [22].

Notwithstanding such successes, it is important to acknowledge that lower tiered surveillance models of care may benefit from further innovation as they tend to be less developed than the higher tier models that involve direct, active care coordination. Recently, technology and automation have supported demonstrations of more advanced clinically-specific surveillance, potentially extending what had previously been available only through utilization-level data. For example, in a program known as Project SONAR [23], an automated survey instrument provided monthly surveillance of patients with Crohn’s disease, alerting a care team when a patient might have experienced symptoms indicative of flare in their underlying disease. The program reported estimated net savings of over 11 percent in the first year for patients who engaged with the survey instrument, mainly due to lower hospitalizations and emergency department visits [23]. Although originally developed as a disease-specific platform, expansion to other clinical segments could support elements of surveillance within a broader context of tiered, adaptive care delivery. Additionally, as health systems recognize the importance of social factors such as family caregiving [24] in managing aging patient populations, opportunities to integrate these resources into tiered engagement models may proliferate.

### Limitations

The present study had several limitations. The study used highly-aggregated Medicare expenditures to obtain attributes that may not be representative of smaller panels of patients under the care of individual providers. Although the method may still be applicable to such smaller panels, the results may vary. Also, the segmentation methodology was based on a spending order index assignment that was intrinsically retrospective in nature, since the spending order remained undefined until after 12 months of beneficiary spending has elapsed. As such, despite indicating potential benefits of tiered care delivery, the method cannot be used directly to prospectively guide operational deployment of care resources in real time. Additionally, the indications for adaptive, tiered care delivery were derived from the single highest index segment of the subpopulation of 10 percent of highest spenders. Although these patients were shown to represent almost half of monthly expenditures, the indicated tiered care may also need to accommodate other lower segment migrations. As indicated earlier, compared to active care coordination models, lower tiered surveillance models of care may be less developed so more research is needed in this area.

## Conclusions

Building upon existing Medicare spending concentration studies, the present study evaluated a new method that segmented high-cost health care expenditures, revealing use patterns to inform care redesign. Our study characterized specific attributes of certain patient subgroups and how they migrate in and out of the highest spending segments. Thus, this new method provided both a general characterization and quantitative parameters of subgroup migration not previously available through usual methods for evaluating spending concentration. The results supported our underlying hypothesis that indiscriminately or persistently providing care coordination or other resources to high-cost beneficiaries could inadequately target care delivery to the subgroups poised to occupy the highest spending segments at any one time. Further support for the hypothesis was provided through cost-effective demonstrations of tiered care delivery models that adaptively placed small high-cost subgroups under active, direct care at any one time, with the remaining majority falling under surveillance levels of care, capable of both meeting baseline needs and detecting the monthly emergence of beneficiaries poised to occupy the highest monthly segments. Additional studies of tiered care delivery are needed both to further explore cost-effectiveness and to extend development of lower tier, surveillance models of care, both of which may be critical to meeting the needs of growing populations of older, medically complex adults.
